# Supplementary material for: Sexual dimorphism in the social behaviour of Cntnap2-null mice correlates with disrupted synaptic connectivity and increased microglial activity in the anterior cingulate cortex
Source: Commun Biol. 2023 Aug 15;6:846. doi: 10.1038/s42003-023-05215-0 (PMC10427688; doi:10.1038/s42003-023-05215-0)
Supplement: Supplementary file 1 — Supplementary Information [file 42003_2023_5215_MOESM1_ESM.pdf]

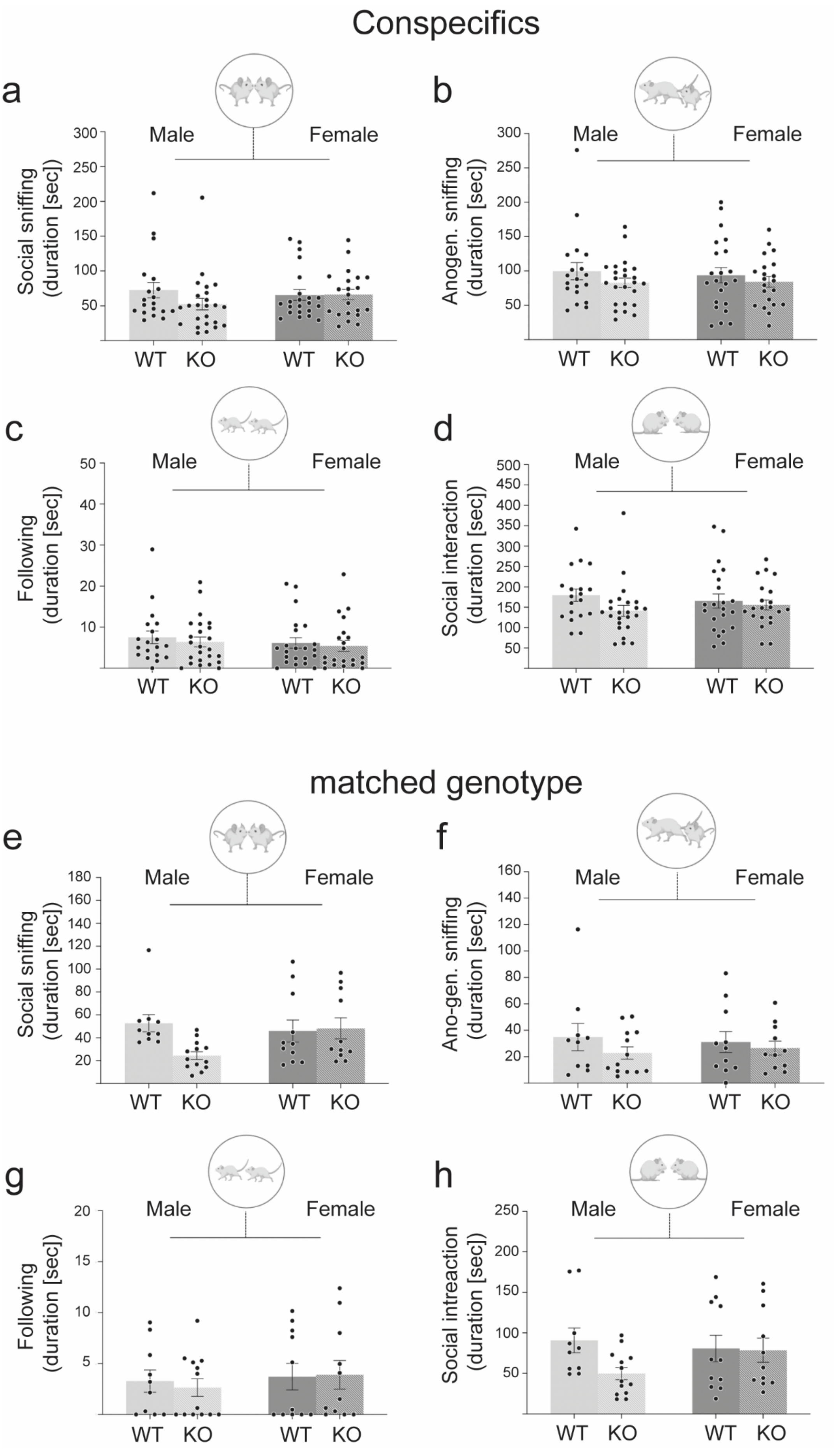
Conspecifics
a
Social sniffing (duration [sec])
Male
Female
WT KO WT KO
b
Anogen. sniffing (duration [sec])
Male
Female
WT KO WT KO
c
Following (duration [sec])
Male
Female
WT KO WT KO
d
Social interaction (duration [sec])
Male
Female
WT KO WT KO
matched genotype
e
Social sniffing (duration [sec])
Male
Female
WT KO WT KO
f
Ano-gen. sniffing (duration [sec])
Male
Female
WT KO WT KO
g
Following (duration [sec])
Male
Female
WT KO WT KO
h
Social intreaction (duration [sec])
Male
Female
WT KO WT KO

Suppl. Fig. 1. No behavioural deficits of juvenile male *Cntnap2* KO mice for duration of interactions. Juvenile mice were analysed in a social interaction assay, in which a test mouse (*Cntnap2* KO or WT mice of either sex) was placed in a novel cage, and a sex- matched juvenile conspecific C57BL/6J mouse **(a-d)** or an age-, sex-, and genotype-matched conspecific **(e-h)** was added. Specifically, social interaction with conspecifics was carried out at P34, and with genotype-matched mice at P38. The duration of their interaction was scored for 10 minutes for social sniffing **(a, e),** ano-genital sniffing **(b, f),** or following **(c, g).** The total interaction frequencies are given in **(d, h)**. Single data points represent individual mice. Statistical analysis was performed using 2-way ANOVA. Symbols above the bars represent an overall effect of genotype (*), sex (+) or interaction (#), while symbols below the chart represent Tukey's *post-hoc* significance between WT and *Cntnap2* KO (*). In each case 1, 2, or 3 symbols represents $p < 0.05$, $p < 0.01$, or $p < 0.001$. There were no differences between juvenile KO mice and WT mice for males or females in any of the behavioural tests. Data presented as means, error bars represent S.E.M. For a-d: n=20-26/group, for e-h: n=10-13/group. Full two-way ANOVA results can be seen in Table S1. Mouse images from Biorender.

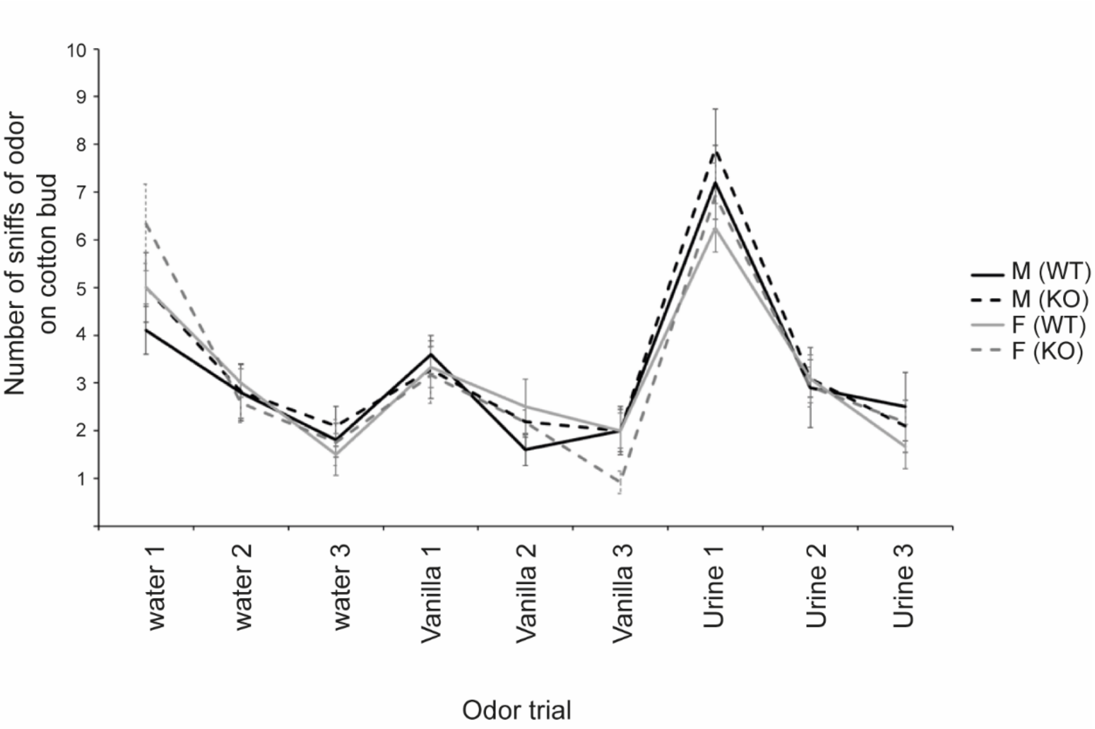

Suppl. Fig 2. Analysis of olfactory habituation/dishabituation in adult *Cntnap2* KO and WT mice of both sexes.

No differences were found in olfactory habituation/dishabituation between KO and WT mice of both sexes, indicating an undisturbed function of the olfactory system. Data presented as means, error bars represent S.E.M. (n=10-12/group). See Materials for details.

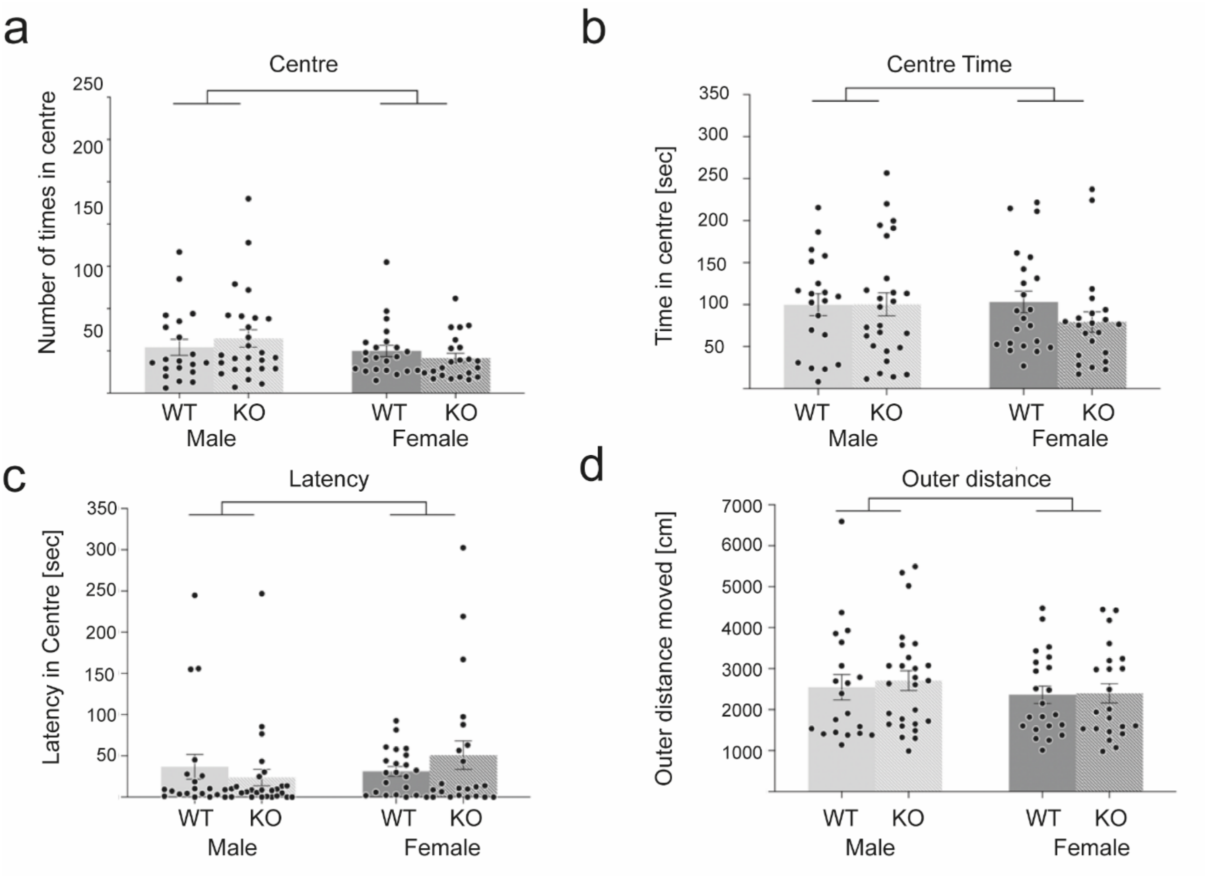

Suppl. Fig. 3. Analysis of anxiety and locomotor activity in male and female juvenile *Cntnap2* KO and WT mice using the Open Field assay.

The mice were analysed at P30. Avoidance of the centre zone was used as a measure of anxiety **(a)** and **(b)**, and latency in centre **(c)** and total distance moved in the outer zone **(d)** were used as a measure of locomotive activity.

For all parameters, there were no differences between juvenile male WT and *Cntnap2* KO mice. Data presented as means, error bars represent S.E.M. (n>20/group). Statistical analysis was performed using 2-way ANOVA. See Materials for details.

a

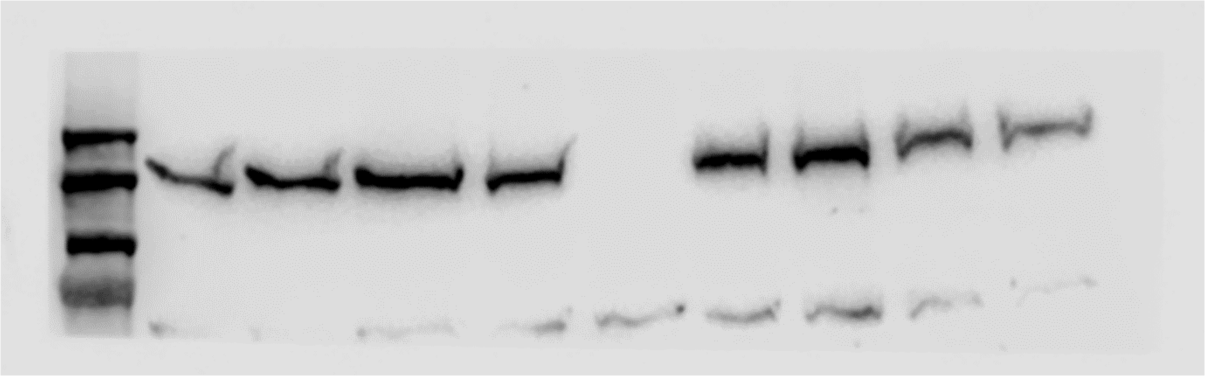

b

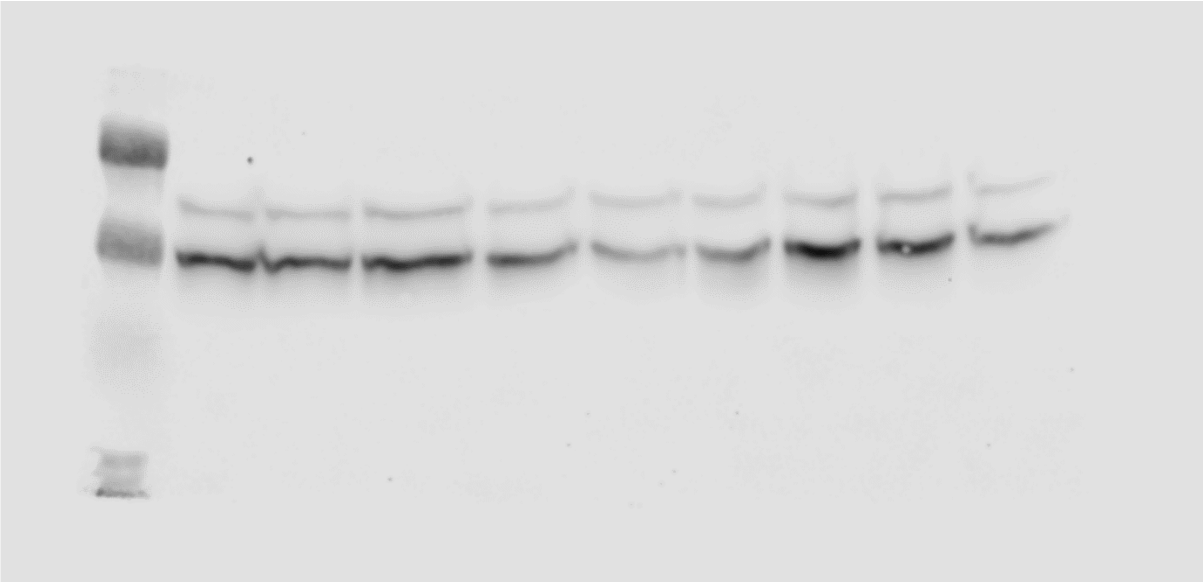

Suppl. Figure 4. Uncropped and un-edited images of blots shown in Fig. 4.

(a) CASPR2 blot

(b) MAPK/ERK blot

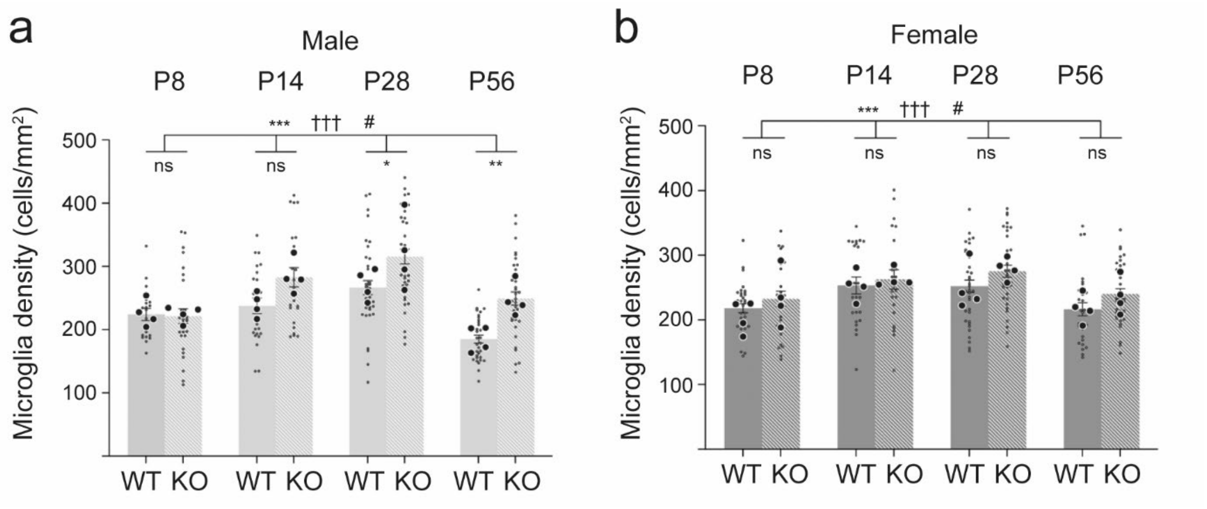

Suppl. Fig. 5. Microglia densities in layer 1 of the ACC of *Cntnap2* KO and WT mice from male and female populations were analysed at four developmental time points P8, P14, P28 and P56.

(a) Microglia cell densities in the male population. There were no differences in cell densities at P8 and P14, but significant increases in KOs compared to WTs at P28 and P56.

(b) In females, there were no significant differences in microglial cell densities at any of the indicated time points.

In all experiments, n=4 KO, n= 4 WT for each timepoint and sex. Large dots represent mice, small dots density on individual sections. Data presented as means, error bars represent S.E.M. Statistical analysis was performed using 2-way ANOVA. Symbols above the bars represent an overall effect of genotype (*), sex (+) or interaction (#), while symbols below the chart represent Tukey's *post-hoc* significance between WT and *Cntnap2* KO (*). In each case 1, 2, or 3 symbols represents $p < 0.05$, $p < 0.01$, or $p < 0.001$.
