# Supplementary material for: Sexual dimorphism in the social behaviour of Cntnap2-null mice correlates with disrupted synaptic connectivity and increased microglial activity in the anterior cingulate cortex
Source: Commun Biol. 2023 Aug 15;6:846. doi: 10.1038/s42003-023-05215-0 (PMC10427688; doi:10.1038/s42003-023-05215-0)
Supplement: Supplementary file 2 — Description of Additional Supplementary Files [file 42003_2023_5215_MOESM2_ESM.pdf]

Description of Additional Supplementary Files

File Name: Supplementary Data 1
Description: Statistical analysis and results for behavioural analysis (referring to Fig. 1 and Suppl. Fig. 1).

File Name: Supplementary Data 2
Description: Statistical analysis and results for GRP-cre analysis (referring to Fig. 2).

File Name: Supplementary Data 3
Description: Statistical analysis and results for spine and bouton analysis (referring to Fig. 3).

File Name: Supplementary Data 4
Description: Statistical analysis and results for microglia analysis (referring to Suppl. Fig. 4, Fig. 5, Fig. 6f, and Fig. 7b).

File Name: Supplementary Data 5
Description: Statistical analysis and results for Western blot and qPCR analysis (referring to Fig. 4b and Fig. 5g).

File Name: Supplementary Data 6
Description: Source data underlying figures.
