# Supplementary material for: Sexual dimorphism in the social behaviour of Cntnap2-null mice correlates with disrupted synaptic connectivity and increased microglial activity in the anterior cingulate cortex
Source: Commun Biol. 2023 Aug 15;6:846. doi: 10.1038/s42003-023-05215-0 (PMC10427688; doi:10.1038/s42003-023-05215-0)
Supplement: Supplementary file 3 — Supplementary Data 1-5 [file 42003_2023_5215_MOESM3_ESM.docx]

| **Supplementary data 1. Behavioural analysis** | | | | | |
| --- | --- | --- | --- | --- | --- |
| **Figure** | **Measurement** | **Means** | **N, number of mice** | **Statistical analysis** | **p-value** |
| **Fig 1A** | **Social sniff  FREQUENCY (conspecifics)**  Male WT vs. KO  Female WT vs. KO | 33.74 vs. 26.96  36.05 vs. 34.43 | 19 vs. 24  22 vs. 21 | **Two-way ANOVA**  Interaction: F (1, 82) = 1.1663  Sex: F (1, 82) = 4.11863  Genotype: F (1, 82) = 3.0856 | p=0.28  ***p=0.04***  p=0.08 |
| **Fig 1B** | **Anogenital sniff  FREQUENCY (conspecifics)**  Male WT vs. KO  Female WT vs. KO | 36.79 vs. 32.08  36.00 vs. 38.19 | 19 vs. 24  22 vs. 21 | **Two-way ANOVA**  Interaction: F (1, 82) = 1.4119  Sex: F (1, 82) = 0.8394  Genotype: F (1, 82) = 0.1879 | p=0.24  p=0.36  p=0.67 |
| **Fig 1C** | **Following  FREQUENCY (conspecifics)**  Male WT vs. KO  Female WT vs. KO | 6.32 vs. 5.33  6.46 vs. 5.71 | 19 vs. 24  22 vs. 21 | **Two-way ANOVA**  Interaction: F (1, 82) = 0.001953  Sex: F (1, 82) = 0.04389  Genotype: F (1, 82) = 0.48225 | p=0.92  p=0.83  p=0.49 |
| **Fig 1D** | **Social interaction FREQUENCY (conspecifics)**  Male WT vs. KO  Female WT vs. KO | 75.45 vs. 63.68  78.50 vs. 78.33 | 19 vs. 24  22 vs. 21 | **Two-way ANOVA**  Interaction: F (1, 82) = 1.937  Sex: F (1, 82) = 3.122  Genotype: F (1, 82) = 2.043 | p=0.17  p=0.08  p=0.16 |
|  |  |  |  |  |  |
| **Fig 1E** | **Social sniffing FREQUENCY (matched genotype)**  Male WT vs. KO  Female WT vs. KO | 42.40 vs. 25.69  35.55 vs. 41.36 | 10 vs. 13  11 vs. 11 | **Two-way ANOVA**  Interaction: F (1, 41) = 17.79  Sex: F (1, 41) = 2.725  Genotype: F (1, 41) = 4.157  ***Tukey's multiple comparisons test:***  M WT vs M KO  M KO vs FKO | ***p<0.001***  p=0.11  ***p=0.05***  ***p<0.001***  ***p<0.001*** |
| **Fig 1F** | **Anogenital sniffing FREQUENCY (matched genotype)**  Male WT vs. KO  Female WT vs. KO | 19.40 vs. 14.46  17.73 vs. 20.19 | 10 vs. 13  11 vs. 11 | **Two-way ANOVA**  Interaction: F (1, 41) = 1.240  Sex: F (1, 41) = 0.3640  Genotype: F (1, 41) = 0.1542 | p=0.27  p=0.55  p=0.70 |
| **Fig 1G** | **Following FREQUENCY (matched genotype)**  Male WT vs. KO  Female WT vs. KO | 3.50 vs. 2.85  4.46 vs. 5.56 | 10 vs. 13  11 vs. 11 | **Two-way ANOVA**  Interaction: F (1, 41) = 0.396  Sex: F (1, 41) = 1.739  Genotype: F (1, 41) = 0.025 | p=0.53  p=0.19  p=0.88 |
| **Fig 1H** | **Social interaction FREQUENCY (matched genotype)**  Male WT vs. KO  Female WT vs. KO | 65.30 vs. 43.00  57.73 vs. 67.00 | 10 vs. 13  11 vs. 11 | **Two-way ANOVA**  Interaction: F (1, 41) = 8.041  Sex: F (1, 41) = 2.177  Genotype: F (1, 41) = 1.369  ***Tukey's multiple comparisons test:***  M WT vs. M KO  M KO vs F KO | ***p=0.007***  p=0.15  p=0.25  ***p=0.03***  ***p=0.02*** |
|  |  |  |  |  |  |
| **Fig S1A** | **Social sniff  DURATION (conspecifics)**  Male WT vs. KO  Female WT vs. KO | 72.48 vs. 52.26  65.61 vs. 66.16 | 19 vs. 24  22 vs. 21 | **Two-way ANOVA**  Interaction: F (1, 82) = 1.439  Sex: F (1, 82) = 0.1653  Genotype: F (1, 82) = 1.293 | p=0.23  p=0.69  p=0.26 |
| **Fig S1B** | **Anogenital sniff  DURATION (conspecifics)**  Male WT vs. KO  Female WT vs. KO | 99.66 vs. 82.75  93.72 vs. 84.19 | 19 vs. 24  22 vs. 21 | **Two-way ANOVA**  Interaction: F (1, 82) = 0.1457  Sex: F (1, 82) = 0.05428  Genotype: F (1, 82) = 0.1.873 | p=0.70  p=0.82  p=0.17 |
| **Fig S1C** | **Following  DURATION (conspecifics)**  Male WT vs. KO  Female WT vs. KO | 7.54 vs. 6.40  6.15 vs. 5.42 | 19 vs. 24  22 vs. 21 | **Two-way ANOVA**  Interaction: F (1, 82) = 0.02297  Sex: F (1, 82) = 0.8022  Genotype: F (1, 82) = 0.4999 | p=0.88  p=0.37  p=0.48 |
| **Fig S1D** | **Social interaction  DURATION (conspecifics)**  Male WT vs. KO  Female WT vs. KO | 179.7 vs. 141.4  165.5 vs. 155.8 | 19 vs. 24  22 vs. 21 | **Two-way ANOVA**  Interaction: F (1, 82) = 0.9457  Sex: F (1, 82) = 0.0002  Genotype: F (1, 82) = 2.675 | p=0.33  p>0.99  p=0.11 |
|  |  |  |  |  |  |
| **Fig S1E** | **Social sniffing  DURATION  (matched genotype)**  Male WT vs. KO  Female WT vs. KO | 52.69 vs. 24.38  45.96 vs. 48.17 | 10 vs. 13  11 vs. 11 | **Two-way ANOVA**  Interaction: F (1, 41) = 4.042  Sex: F (1, 41) = 1.265  Genotype: F (1, 41) = 2.956 | p=0.05  p=0.27  p=0.09 |
| **Fig S1F** | **Anogenital sniffing DURATION (matched genotype)**  Male WT vs. KO  Female WT vs. KO | 34.86 vs. 22.81  31.13 vs. 26.56 | 10 vs. 13  11 vs. 11 | **Two-way ANOVA**  Interaction: F (1, 41) = 0.2819  Sex: F (1, 41) = 0.00003  Genotype: F (1, 41) = 1.393 | p=0.60  p>0.99  p=0.24 |
| **Fig S1G** | **Following  DURATION (matched genotype)**  Male WT vs. KO  Female WT vs. KO | 3.28 vs. 2.64  3.71 vs. 3.90 | 10 vs. 13  11 vs. 11 | **Two-way ANOVA**  Interaction: F (1, 41) = 0.1253  Sex: F (1, 41) = 0.5189  Genotype: F (1, 41) = 0.03706 | p=0.73  p=0.48  p=0.85 |
| **Fig S1H** | **Social interaction  DURATION (matched genotype)**  Male WT vs. KO  Female WT vs. KO | 90.83 vs. 49.83  80.81 vs. 78.63 | 10 vs. 13  11 vs. 11 | **Two-way ANOVA**  Interaction: F (1, 41) = 2.064  Sex: F (1, 41) = 0.4827  Genotype: F (1, 41) = 2.552 | p=0.16  p=0.49  p=0.12 |

| **Supplementary data 2. GRP-cre analysis** | | | | | |
| --- | --- | --- | --- | --- | --- |
| **Figure** | **Measurement** | **Means** | **N, number of mice (number of hemispheres)** | **Statistical analysis** | **p-value** |
| **Fig 2C** | **Layer2/3**  GRP-Cre+ WT vs. KO  CUX1 WT vs. KO | 6.45 vs. 6.3  31.01 vs. 35.71 | 4 (8) vs. 4 (8)  4 (8) vs. 4 (8) | **2-tailed Unpaired t tests:**  t=0.102, df=14  t=1.075, df=14 | p=0.92  p=0.30 |
| **Fig 2D** | **GRP-Cre % CUX1**  GRP-Cre+ WT vs. KO | 21.48 vs. 18.23 | 4 (8) vs. 4 (8) | **2-tailed Unpaired t test:**  t=0.666, df=14 | p=0.52 |
| **Fig 2F** | **Layer 5**  GRP-Cre+ WT vs. KO  CTIP2 WT vs. KO | 5.69 vs. 4.43  49.18 vs. 48.19 | 4 (8) vs. 4 (8)  4 (8) vs. 4 (8) | **2-tailed Unpaired t tests:**  t=0.932, df=15  t=0.144, df=15 | p=0.92  p=0.30 |
| **Fig 2G** | **GRP-Cre % CTIP2**  GRP-Cre+ WT vs. KO | 10.24 vs. 9.90 | 4 (8) vs. 4 (8) | **2-tailed Unpaired t test:**  t=0.205, df=15 | p=0.84 |

| **Supplementary data 3. Spine & bouton analysis** | | | | | |
| --- | --- | --- | --- | --- | --- |
| **Figure** | **Measurement** | **Means** | **N, number of mice (number of dendrites/ sections)** | **Statistical analysis** | **p-value** |
| **Figs 3A-D** | **Spine densities in ACC**  Male  P8 WT vs. KO  P14 WT vs. KO  P28 WT vs. KO  P56 WT vs. KO  Female  P8 WT vs. KO  P14 WT vs. KO  P28 WT vs. KO  P56 WT vs. KO | 0.359 vs. 0.320  0.487 vs. 0.369  0.540 vs. 0.391  0.468 vs. 0.469  0.340 vs. 0.322  0.508 vs. 0.485  0.557 vs. 0.554  0.530 vs. 0.521 | 4 (29) vs. 4 (42)  4 (33) vs. 4 (29)  4 (33) vs. 4 (32)  4 (33) vs. 4 (33)  4 (36) vs. 4 (26)  4 (38) vs. 4 (37)  4 (48) vs. 4 (48)  4 (47) vs. 4 (48) | **Three-way ANOVA**  Age: F (3, 576) = 99.76  Sex: F (1, 576) = 29.26  Genotype: F (1, 576) = 25.06  Age x Sex: F (3, 576) = 5.84  Age x Genotype: F (3, 576) = 3.86  Sex x Genotype: F (1, 576) = 13.47  Age x Sex x Genotype: F (3, 576) = 3.66  ***Tukey’s multiple comparisons test:***  P14 M WT vs. P14 M KO  P28 M WT vs. P28 M KO  P14 M KO vs. P14 F KO  P28 M KO vs. P28 F KO  P8 M WT vs P14 M WT  P8 M WT vs P28 M WT  P8 M WT vs P28 M WT  P8 M KO vs P56 M KO  P14 M KO vs P56 M KO  P8 F WT vs P14 F WT  P8 F WT vs P28 F WT  P8 F WT vs P56 F WT  P8 F KO vs P14 F KO  P8 F KO vs P28 F KO  P8 F KO vs P56 F KO | ***p<0.001***  ***p<0.001***  ***p<0.001***  ***p<0.001***  ***p=0.009***  ***p<0.001***  ***p=0.01***  ***p<0.001***  ***p<0.001***  ***p<0.001***  ***p<0.001***  ***p<0.001***  ***p<0.001***  ***p<0.001***  ***p<0.001***  ***p=0.002***  ***p<0.001***  ***p<0.001***  ***p<0.001***  ***p<0.001***  ***p<0.001***  ***p<0.001*** |
| **Fig 3G** | **Spine densities in M2**  Male: P14 WT vs. KO  Female: P14 WT vs. KO | 0.336 vs. 0.361  0.340 vs. 0.380 | 4 (43) vs. 4 (42)  4 (41) vs. 4 (44) | **Two-way ANOVA**  Interaction: F (1, 166) = 0.295  Sex: F (1, 166) = 0.747  Genotype: F (1, 166) = 5.471  ***Tukey's multiple comparisons test:***  *No significance for genotype* | p=0.59  p=0.39  ***p=0.02*** |
| **Fig 3H** | **Pre-synaptic bouton (VGLUT1) volume, P14**  M WT vs. KO  F WT vs. KO | 0.050 vs. 0.030  0.054 vs. 0.050 | 3 (15) vs. 3 (15)  3 (15) vs. 3 (15) | **Two-way ANOVA**  Interaction: F (1, 56) = 2.258  Sex: F (1, 56) = 5.940  Genotype: F (1, 56) = 5.421  ***Tukey's multiple comparisons test:***  Male WT vs. Male KO  Male KO vs Female KO | p=0.14  ***p=0.02***  ***p=0.02***  ***p<0.05***  ***p=0.04*** |

| **Supplementary data 4. Microglia analysis** | | | | | |
| --- | --- | --- | --- | --- | --- |
| **Figure** | **Measurement** | **Means** | **N, Number of mice (number of microglia)** | **Statistical analysis** | **p-value** |
| **Fig S4** | **Microglia cell density in layer 1 of ACC**  Male  P8 WT vs. KO  P14 WT vs. KO  P28 WT vs. KO  P56 WT vs. KO  Female  P8 WT vs. KO  P14 WT vs. KO  P28 WT vs. KO  P56 WT vs. KO | 224.4 vs 221.5  237.8 vs. 282.9  266.6 vs. 315.6  185.1 vs. 249.4  218.0 vs. 232.7  253.3 vs. 262.8  252.2 vs. 275.2  216.5 vs. 240.3 | 4 (19) vs. 4 (30)  4 (24) vs. 4 (24)  4 (37) vs. 4 (37)  4 (31) vs. 4 (35)  4 (32) vs. 4 (25)  4 (23) vs. 4 (23)  4 (36) vs. 4 (36)  4 (29) vs. 4 (35) | **Three-way ANOVA**  Age: F (3, 460) = 26.91  Sex: F (1, 460) = 0.053  Genotype: F (1, 460) = 26.61  Age x Sex: F (3, 460) = 2.75  Age x Genotype: F (3, 460) = 2.18  Sex x Genotype: F (1, 460) = 7.72  Age x Sex x Genotype: F (3, 460) = 1.34  ***Tukey’s multiple comparisons test:***  P28 M WT vs. P28 M KO  P56 M WT vs. P56 M KO  P8 M KO vs. P14 M KO  P8 M KO vs. P28 M KO  P28 M KO vs. P56M KO  P28 M WT vs. P56 M WT | ***p<0.001***  p=0.47  ***p<0.001***  ***p=0.04***  p=0.09  p=0.05  p=0.26  ***p=0.03***  ***p<0.001***  ***p=0.01***  ***p<0.001***  ***p=0.03***  ***p<0.001*** |
| **Fig 5B** | **Microglia branch points**  Male  P8 WT vs. KO  P14 WT vs. KO  Female  P8 WT vs. KO  P14 WT vs. KO | 57.05 vs. 38.33  47.94 vs. 30.75  62.55 vs. 56.05  60.78 vs. 53.19 | 4 (21) vs. 4 (24)  4 (31) vs. 4 (28)  4 (20) vs. 4 (22)  4 (23) vs. 4 (32) | **Three-way ANOVA**  Genotype: F (1, 193) = 19.440  Age: F (1, 193) = 3.534  Sex: F (1, 193) = 26.610  Genotype x Age: F (1, 193) = 0.001  Genotype x Sex: F (1, 193) = 3.695  Age x Sex: F (1, 193) = 1.133  Age x Sex x Genotype: F (1, 193) = 0.053  ***Tukey’s multiple comparisons test:***  P8 M WT vs. P8 WT KO  P14 M WT vs. P14 M KO  P14 M KO vs. P14 F KO | ***p<0.001***  p=0.06  ***p<0.001***  p=0.97  p=0.06  p=0.29  p=0.82  ***p=0.04***  ***p=0.02***  ***p<0.001*** |
| **Fig 5F** | **Microglia branch level**  Male  P8 WT vs. KO  P14 WT vs. KO  Female  P8 WT vs. KO  P14 WT vs. KO | 8.71 vs. 6.75  7.03 vs. 6.57  8.10 vs. 7.86  7.91 vs. 6.91 | 4 (21) vs. 4 (24)  4 (30) vs. 4 (28)  4 (20) vs. 4 (22)  4 (23) vs. 4 (32) | **Three-way ANOVA**  Genotype: F (1, 192) = 10.610  Age: F (1, 192) = 7.112  Sex: F (1, 192) = 2.315  Genotype x Age: F (1, 192) = 0.422  Genotype x Sex: F (1, 192) = 1.103  Age x Sex: F (1, 192) = 10.403  Age x Sex x Genotype: F (1, 192) = 4.071  ***Tukey’s multiple comparisons test***  P8 WT vs. KO  P8 M WT vs. P14 M WT | ***p<0.001***  ***p=0.008***  p=0.13  p=0.52  p=0.29  p=0.53  ***p<0.05***  ***p=0.01***  ***p=0.04*** |
| **Fig 5C** | **Microglia dendrite length**  Male  P8 WT vs. KO  P14 WT vs. KO  Female  P8 WT vs. KO  P14 WT vs. KO | 468.5 vs. 335.8  381.6 vs. 285.2  476.8 vs. 422.0  489.3 vs. 429.1 | 4 (21) vs. 4 (24)  4 (30) vs. 4 (28)  4 (20) vs. 4 (22)  4 (23) vs. 4 (32) | **Three-way ANOVA**  Genotype: F (1, 192) = 17.670  Age: F (1, 192) = 2.081  Sex: F (1, 192) = 17.890  Genotype x Age: F (1, 192) = 0.141  Genotype x Sex: F (1, 192) = 1.945  Age x Sex: F (1, 192) = 3.686  Age x Sex x Genotype: F (1, 192) = 0.260  ***Tukey’s multiple comparisons test***  P8 M WT vs. P8 M KO  P14 M KO vs. P14 F KO | ***p<0.001***  p=0.15  ***p<0.001***  p=0.71  p=0.16  p=0.06  p=0.61  ***p=0.03***  ***p=0.002*** |
| **Fig 5D** | **Microglia primary processes**  Male  P8 WT vs. KO  P14 WT vs. KO  Female  P8 WT vs. KO  P14 WT vs. KO | 4.71 vs. 3.50  5.50 vs. 4.25  4.70 vs. 4.41  5.44 vs. 5.63 | 4 (21) vs. 4 (24)  4 (30) vs. 4 (28)  4 (20) vs. 4 (22)  4 (23) vs. 4 (32) | **Three-way ANOVA**  Genotype: F (1, 192) = 11.450  Age: F (1, 192) = 21.160  Sex: F (1, 192) = 8.461  Genotype x Age: F (1, 192) = 0.345  Genotype x Sex: F (1, 192) = 9.726  Age x Sex: F (1, 192) = 0.300  Age x Sex x Genotype: F (1, 192) = 0.465  ***Tukey’s multiple comparisons test***  P8 M WT vs. P8 M KO  P14 M WT vs. P14 M KO  P8 F KO vs P14 F KO  P14 M KO vs. P14 F KO | ***p<0.001***  ***p<0.001***  ***p=0.004***  p=0.56  ***p=0.002***  p=0.58  p=0.50  ***p=0.03***  ***p=0.005***  ***p=0.01***  ***p=0.001*** |
| **Fig 5E** | **Microglia volume in layer 1 of ACC**  Male  P8 WT vs. KO  P14 WT vs. KO  Female  P8 WT vs. KO  P14 WT vs. KO | 643.2 vs. 517.8  481.3 vs. 343.1  644.9 vs. 698.8  487.7 vs. 468.8 | 4 (21) vs. 4 (24)  4 (30) vs. 4 (28)  4 (20) vs. 4 (22)  4 (23) vs. 4 (32) | **Three-way ANOVA**  Genotype: F (1, 192) = 3.262  Age: F (1, 192) = 32.670  Sex: F (1, 192) = 6.176  Genotype x Age: F (1, 192) = 0.457  Genotype x Sex: F (1, 192) = 5.558  Age x Sex: F (1, 192) = 0.160  Age x Sex x Genotype: F (1, 192) = 0.224  ***Tukey’s multiple comparisons test***  P8 F KO vs P14 F KO | p=0.07  ***p<0.001***  ***p=0.01***  p=0.50  ***p=0.02***  p=0.69  p=0.64  ***p=0.006*** |
| **Fig 6D** | **IBA1/VGLUT1 coloc, P**  Male: P14 WT vs. KO  Female: P14 WT vs. KO | 0.00156 vs. 0.00124  0.00124 vs. 0.00148 | 3 (23) vs. 3 (22)  3 (22) vs. 3 (25) | **Two-way ANOVA**  Interaction: F (1, 88) = 2.422  Sex: F (1, 88) = 8.379  Genotype: F (1, 88) = 11.07  ***Tukey’s multiple comparisons test:***  P14 M WT vs. P14 M KO  P14 M KO vs P14 F KO | p=0.12  ***p=0.005***  ***p=0.001***  ***p=0.01***  ***p=0.004*** |
| **Fig 6F** | **Total % coloc**  WT vs. KO | Median  35.14 vs. 44.38 | 3 vs. 3 | **Two-tailed Mann-Whitney test:**  Mann-Whitney U = 087884 | ***p<0.001*** |
| **Fig 7B** | **Microglia cell density in layer 1 of ACC: minocycline, p17**  Male: P17 WT vs. KO  Male: P26 WT vs. KO | 247.1 vs. 152.3  215.5 vs. 140.8 | 4 (8) vs. 4 (8)  4 (8) vs. 4 (8) | **2-tailed Unpaired t tests:**  t=3.150, df=22  t=2.361, df=22 | ***p=0.005***  ***p=0.03*** |

| **Supplementary data 5. Western blot and qPCR analysis** | | | | | |
| --- | --- | --- | --- | --- | --- |
| **Figure** | **Measurement** | **Means** | **N, number of mice** | **Statistical analysis** | **p-value** |
| **Fig 4B** | **Caspr2, P28**  P28 M WT Male vs F WT | 0.021 vs. 0.024 | 4 vs. 4 | **Unpaired, two-way t-test**  t = 0.3440, df = 6 | p=0.74 |
| **Fig 5G** | **P2Y6**  M WT vs. KO  F WT vs. KO | 0.01611 vs. 0.19936  0.01705 vs. 0.01571 | 3 vs. 3  3 vs. 3 | **Two-way ANOVA**  Interaction: F (1, 276) = 47.210  Sex: F (1, 276) = 16.450  Genotype: F (1, 276) = 8.273  ***Tukey's multiple comparisons test:***  P14 M WT vs. P14 M KO  P14 F KO vs. P14 F WT  P14 M KO vs P14 F KO | ***p<0.001***  ***p<0.001***  ***p=0.004***  ***p<0.001***  ***p=0.03***  ***p<0.001*** |
